# Impact of the Applied Electrode System on Properties of Electrodeposited Calcium Phosphate Coatings

**DOI:** 10.3390/ma18030539

**Published:** 2025-01-24

**Authors:** Klaudia Iwaniak, Witold Kaczorowski, Barbara Burnat, Jacek Grabarczyk

**Affiliations:** 1 Institute of Material Science and Engineering, Lodz University of Technology, 1/15 Stefanowskiego Str., 90-537 Lodz, Poland; witold.kaczorowski@p.lodz.pl (W.K.);; 2 University of Lodz, Faculty of Chemistry, Department of Inorganic and Analytical Chemistry, 12 Tamka Str., 91-403 Lodz, Poland

**Keywords:** electrochemical deposition, calcium phosphate, hydroxyapatite, coatings, titanium alloys, Ti6Al7Nb alloy

## Abstract

The morphology and physicochemical properties of electrochemically deposited CaP coatings depend on the applied process parameters; however, the influence of different electrode systems has not been studied so far. In this work, the possibility of electrochemical deposition of CaP coatings on Ti6Al7Nb alloy using different electrode systems (two-electrode and three-electrode) and the influence of the electrode system and selected ranges of deposition parameters on the properties of the deposited CaP coatings were investigated. The morphology and physicochemical properties of the CaP coatings were characterized by scanning electron microscopy (SEM), energy dispersive X-ray spectroscopy (EDS), Raman spectroscopy, X-ray diffraction (XRD), and corrosion studies. The results confirmed the effective electrodeposition of CaP coatings using both electrode systems. The applied electrode system and deposition parameters cause changes in the morphology of the obtained coatings. Chemical structure analysis confirmed the presence of mainly hydroxyapatite in the deposited CaP coatings. With the change in voltage/potential in a more cathodic direction, in addition to hydroxyapatite, a dicalcium phosphate dihydrate (DCPD) structure appears. The corrosion tests have shown that the applied deposition parameters have an impact on corrosion resistance and the deposited coatings exhibited protective properties against corrosion under physiological conditions. The CaP coatings with optimal properties for biomedical applications were deposited at a voltage of −4 V in the two-electrode system and a potential of −4 V_SCE_ in the three-electrode system.

## 1. Introduction

Titanium and its alloys are commonly used biomaterials as orthopedic and dental implants or implantological elements related to bone surgery, i.e., plates or screws [1]. These materials are characterized by good biocompatibility, corrosion resistance, and adequate mechanical strength [2]. However, among patients with implanted metallic material, negative effects on the body have been observed [3]. This includes allergic reactions to alloying elements, such as vanadium or aluminum [3,4,5]. In addition, toxic ions may be released into the surrounding tissues, causing inflammation or metallosis [6,7,8]. Another important aspect is implant loosening, which occurs due to poor integration with the bone. This can lead to the deposition of wear products into the tissue area and even bone resorption [9,10,11]. Moreover, titanium and its alloys are incapable of properly stimulating bone cell proliferation [12]. Solutions are being sought to overcome these problems.

Today’s research focuses on increasing the functionality of metallic biomaterials based on surface modification [13,14,15,16,17]. The implantable biomaterial must be biocompatible, which means that inflammation or the body’s response to the foreign material should not occur [18,19,20]. Coatings based on bioceramics, which can actively participate in bone regeneration, seem to be the best choice for further surface modification of titanium implants. For this reason, calcium phosphates (CaPs) may be an ideal solution [21]. CaP coatings can significantly improve implant–tissue interaction and provide bioactive properties without interfering with the properties of the substrate [5,22]. CaP as a bioactive coating on the metallic surface of the implant may exhibit osteoinduction, understood as the ability to stimulate the growth of bone tissue [23,24]. Furthermore, CaPs ensure proper cell proliferation and osseointegration, which determines the stability and integrity of the implant with the bone. This is due to the similarity of their chemical structure to hard tissues [20,24,25]. Some CaPs belong to resorbable bioceramics, which gradually degrade in a time frame and allow for the replacement by bone tissue [26,27,28]. However, despite many advantages, CaP-based biomaterials have poor mechanical properties; therefore, their use in highly stressed locations is limited [26,29,30]. Thus, by coating titanium substrates with CaP coatings, a combination of the benefits of both materials can be achieved, where the mechanical strength of the metal is combined with the bioactive properties of CaPs, promoting a faster healing process and integration with tissues [31,32]. Additionally, by covering titanium alloy with a CaP coating, it is possible to obtain a barrier against the release of toxic ions of elements from the alloy into the body [33,34].

CaPs include a number of compounds that differ in their properties and possible applications in biomedicine, such as α-tricalcium phosphate (α-TCP), β-tricalcium phosphate (β-TCP), dicalcium phosphate dihydrate also called brushite (DCPD), amorphous calcium phosphate (ACP), octacalcium phosphate (OCP), and hydroxyapatite (HAp) [24,35]. Hydroxyapatite, due to its chemical analogy to the mineral component of human bones, is the most frequently used compound from this group [20,28,36,37]. HAp is of wide interest in the fields of biomedicine in various applications, where it can contribute to the regeneration or replacement of bone tissue [25,36,37].

CaP coatings are commercially produced by plasma spraying [38,39,40,41]. However, due to the frequent appearance of an amorphous CaP structure, relatively rough process conditions, and line-of-sight process, alternative methods of obtaining CaP coatings are subject to research [38,42,43]. Coatings can be obtained also using other methods, including sol–gel [44,45,46], electrophoretic deposition [47,48], magnetron sputtering [49,50], electrochemical deposition [51,52,53,54], etc. Nowadays, many studies focus on the method of electrochemically assisted deposition of CaP coatings, due to the low process temperatures, the possibility of complex modification of the substrate, and the ability to control the parameters of the deposition process [54,55,56]. Controlling the process parameters allows to adjust the chemical structure of the deposited coatings and adapt the obtained coatings to the required applications [54,55,56]. Despite many advantages of the electrodeposition process, the obtained coatings are often not chemically homogeneous. In addition to or instead of HAp, other forms of CaPs are produced, such as DCPD, OCP, ACP, or CDHA, i.e., calcium-deficient hydroxyapatite [57,58]. Therefore, it is essential to optimize the process conditions to achieve coatings with the desired properties [54].

The properties of calcium phosphate coatings may depend on controllable parameters of the electrodeposition processes, such as voltage, potential, current density, process duration, deposition temperature, and electrolyte pH. Nevertheless, the electrode system set-up used in the electrochemical deposition of CaP coatings also seems to be a key aspect. During the electrodeposition process, the charge of the cathode (working electrode) changes as a result of nucleation and growth of CaPs on the modified surface. Therefore, in a two-electrode system, it is not possible to easily control and maintain constant electrochemical conditions, despite maintaining the applied voltage or current using a power supply. This is due to the fact that CaP deposition could cause passivation of the cathode, which contributes to the change in charge on the working electrode. The use of a three-electrode system allows for maintaining the applied potential or current density with respect to the reference electrode, e.g., calomel or silver chloride electrode. It is, therefore, possible to avoid the occurring charge changes on the cathode, and thus maintain a constant deposition rate throughout the process [59].

Therefore, in this work, attempts were made to deposit a homogeneous hydroxyapatite coating on a Ti6Al7Nb alloy substrate using the electrochemical method with various parameter ranges and electrode systems. The main goal of the research is to analyze the impact of using a different electrode system on the morphology and structure of the coatings, which has not been compared so far. Given that specific ranges of deposition voltage/potential are utilized in the chosen electrode systems, it is crucial to evaluate their impact on the properties of the coatings. Additionally, there are limited literature reports on the electrochemical deposition of CaP coatings on Ti6Al7Nb titanium alloy, a more biocompatible alternative to Ti6Al4V alloy, which contains harmful vanadium ions. Since the substrate involved in the modification process can significantly affect the electrochemical deposition results, it is important to optimize the CaP electrodeposition parameters using a specific alloy [54].

## 2. Materials and Methods

### 2.1. Material

Ti6Al7Nb titanium alloy discs (Medgal Ltd., Księżyno, Poland) of 16 mm diameter and 6 mm thickness were used as substrates for CaP coatings. The discs were ground with 120- to 2500-grit sandpaper and then polished with a colloidal silica suspension. In the next stage, the samples were washed in ethanol at 30 °C for 20 min using an ultrasonic bath. Before the processing, the substrates were washed with distilled water.

### 2.2. Electrochemical Coatings Deposition

Electrolytes contained 0.042 M Ca(NO_3_)_2_ and 0.025 M NH_4_H_2_PO_4_ (Chempur, Piekary Śląskie, Poland; both). The salts were Ca and P precursors, respectively. The applied Ca/P molar ratio was equal to 1.68, which corresponds to the stoichiometric hydroxyapatite.

**Figure 1:**
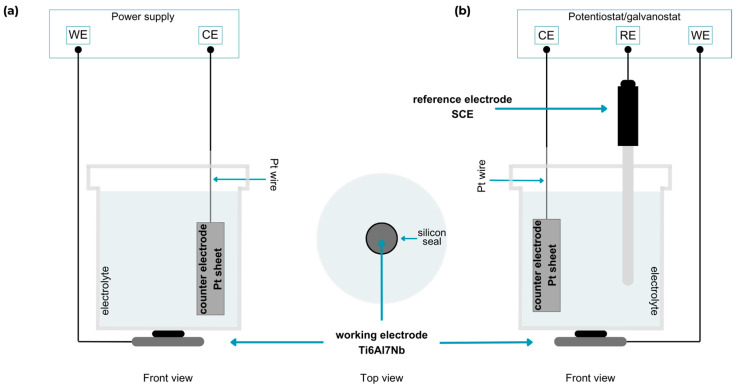
Schematic illustration of the set-up for (**a**) two-electrode system and (**b**) three-electrode system of electrochemical deposition (created using canva.com [60]).

The electrochemical processes were carried out in two variants: using the ITECH IT6720 power supply (ITECH Electronic Co., Ltd., Taipei, Taiwan) in a standard two-electrode system and the Autolab PGSTAT 302N potentiostat-galvanostat operated by NOVA 1.11 software (Metrohm, Autolab, Utrecht, The Netherlands) in a three-electrode system. The titanium alloy was the working electrode (WE; with an exposed area of 0.79 cm^2^), the platinum sheet was the counter electrode (CE), and in the three-electrode system, a saturated calomel electrode (SCE, EUROSENSOR, Gliwice, Poland) was additionally used as the reference electrode (RE) (Figure 1). Cathodic processes in both variants were carried out in the constant cell voltage (two-electrode) and potentiostatic mode (three-electrode). Different constant voltages from −4 V to −6 V in the two-electrode system and constant potential from −4 V_SCE_ to −6 V_SCE_ in the three-electrode system were used, where the deposition time was 2 h for both set-up systems (Table 1). After electrochemical deposition, the coated samples were washed with distilled water and left to dry in a chamber at 36 °C. The coating deposition rates in the two-electrode system ranged from 0.017 to 0.038 µm/min and in the three-electrode system from 0.075 to 0.175 µm/min. The rates were calculated based on the coating thicknesses and their deposition time.

### 2.3. Coatings Characterization Methods

After surface modifications, the samples were subjected to observations of their surface morphology using a scanning electron microscope (SEM). The composition and chemical structure were determined by energy-dispersive X-ray spectroscopy (EDS), Raman spectroscopy, and X-ray diffraction measurements (XRD). The corrosion tests were aimed at verifying the corrosion resistance of substrates with deposited coatings.

The morphology of the calcium phosphate coatings deposited on the Ti6Al7Nb alloy was analyzed using a scanning electron microscope JEOL JSM-6610LV (JEOL, Tokyo, Japan). Surface morphology images were obtained using secondary electron detection (SEI) mode. Observations were performed using high vacuum, at electron beam acceleration voltages of 10 kV and 20 kV. The images were taken at magnifications of 100×, 250×, and 800×.

The Hommel-Etamic W20 profilometer (Jenoptik, Jena, Germany) was used to determine the surface roughness of the CaP coatings. Based on the analysis, the average values of coating roughness were determined.

Energy dispersive X-ray spectroscopy studies of the chemical composition were also performed using the apparatus mentioned above, which additionally has a built-in X-Mac 80 X-ray microanalysis system (X-Max 80 EDS, Oxford Instruments, Oxford, UK).

Studies using X-ray diffraction (XRD) made it possible to determine the phase composition of the tested coatings. The diffraction tests were performed on an Empyrean X-ray diffractometer (Panalytical Empyrean, Almeo, The Netherlands). The X-ray source was a Co tube working at an accelerating voltage of 40 kV and with a current value of 40 mA. Analysis of diffraction patterns was made with the use of High Score Plus software (ver. 3.0 e, Panalytical Empyrean, Almeo, The Netherlands) and ICDD PDF 4+ database.

Raman spectroscopy studies were performed to determine the chemical structure of the coatings. Raman signals (spectra and maps) were collected with an InVia Raman microscope (Renishaw plc, Gloucestershire, UK). The CaP coatings were analyzed using a 532 nm laser beam with a 50× objective lens (Carl-Zeiss, Jena, Germany). Single spectra were collected in the spectral collection range 300–1500 cm^−1^. Raman maps were collected from 50 μm × 50 μm areas with 2 µm spatial resolution. The operations on the data (baseline correction), multi-file data (peak area, ratio map), and direct classical least squares (DCLS) component analysis were performed using the Wire 5.5 application. The maps obtained show the homogeneity of the chemical structure of the produced coatings and, above all, the influence of the deposition processes on the concentration of the amorphous structures in the coatings. Based on the deconvolution of the peak at about 963 cm**^−^**^1^ into two peaks: the first at about 950 cm**^−^**^1^ (characteristic of the amorphous calcium phosphate phase), the second at about 963 cm**^−^**^1^ (characteristic of the crystalline HAp phase), the ratio of their areas I_950_/I_963_ was determined, and then the homogeneity of the chemical structure of the deposited coatings was graphically presented on the maps. The deconvolution process for the selected spectrum is presented in Figure S1 in Supplementary Materials. Furthermore, for coatings that showed the presence of a DCPD phase in XRD studies, DCLS mapping was performed to demonstrate the distribution of this phase on the surface. For this purpose, the spectra for DCPD (Figure S2) and HAp (Figure S3) were used. Each time, the look-up table (LUT) was set to obtain a view of a 5–95% signal.

The corrosion resistance of the modified samples was evaluated by means of potentiodynamic polarization (PP). Corrosion tests were performed in Phosphate-Buffered Saline (PBS) solution (pH 7.4, 37 °C, deaerated with argon) using a potentiostat/galvanostat Autolab PGSTAT30 (EcoChimie, Utrecht, The Netherlands). The set-up featured a three-electrode system: the working electrode was the test sample (exposed area of 0.64 cm^2^), platinum mesh served as the counter electrode, and a saturated calomel electrode (SCE) as the reference. Samples were stabilized for 1800 s in open circuit conditions before polarization. PP curves ranged from −0.4 V to +1 V (vs. SCE) and were registered with a scan rate of 1 mV/s. Corrosion data were processed with CView software (version 3.2b; Scribner Associates Inc., Southern Pines, NC, USA).

## 3. Results and Discussion

### 3.1. Scanning Electron Microscopy

The surface morphology of the coatings deposited at various voltages/potentials has been assessed through the SEM images of the coatings.

**Figure 2:**
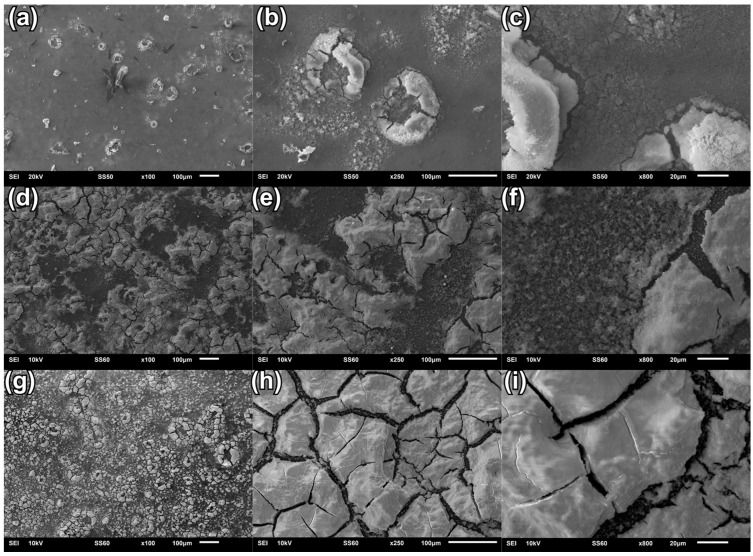
SEM images of the coatings deposited by electrochemical method using a two-electrode system at constant cell voltage (**a**–**c**) −4 V; (**d**–**f**) −5 V; (**g**–**i**) −6 V.

Figure 2 presents the image comparison of the surface morphology of the CaP coatings electrochemically deposited at different deposition voltages using a two-electrode set-up system. It can be stated that the coating deposited at −4 V is relatively smooth and compact, compared to coatings deposited at other voltages in a two-electrode system (Figure 2a–c). A few effects, like microcracks and plate-like CaP elements (Figure 2a,b), are visible due to hydrogen release during the process, which can lead to a break in the continuity of the CaP coating [51,54]. Microcracks may also be the result of stress differences between the substrate and the coating. As a result of applying a voltage of −5 V, the surface of the coating becomes more developed, with additional micropores and cracks appearing (Figure 2d–f). In the structure of this coating, one can observe the most developed micropores, which are the result of hydrogen release during the deposition of the coating. Electrochemical deposition of a CaP coating at a voltage of −6 V ensures a relatively thicker coating compared to coatings deposited at other voltages in a two-electrode system. The obtained coating shows microcracks with irregular grain shapes (Figure 2h,i). The cracks are structurally similar to those formed when the coating was deposed at −5 V, but in this case, uniformly cover the substrate. In some places, there are larger build-ups (Figure 2g). Regardless of the deposition voltage, no homogeneous coatings can be obtained. Furthermore, by changing the voltage towards a more cathodic direction, the surface of the CaP coating becomes more developed.

**Figure 3:**
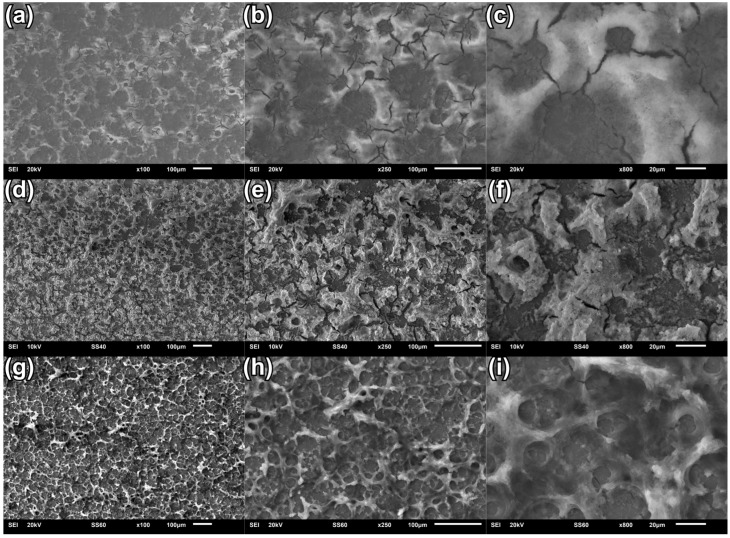
SEM images of the coatings deposited in the potentiostatic mode using a three-electrode system at (**a**–**c**) −4 V_SCE_; (**d**–**f**) −5 V_SCE_; (**g**–**i**) −6 V_SCE._

The effects of CaP coatings’ electrodeposition using a three-electrode system are presented in Figure 3. The electrochemical deposition carried out at the potential value of −4 V_SCE_ ensures compact substrate coverage with small cracks (Figure 3a–c). The coating does not have developed micropores and appears to thickly cover the substrate. The CaP coating deposited at −5 V_SCE_ is characterized by a more developed morphology with a larger number of micropores compared to the coating deposited at −4 V_SCE_ (Figure 3d–f). Micropores are probably the result of hydrogen co-evolution during the process [61]. This coating also shows the most numerous microcracks. As the deposition potential change to −6 V_SCE_, the deposited coating is characterized by a developed porous structure, and it can also be stated that this coating is much more homogeneous and covers the entire surface compared to the coating deposited at −5 V_SCE_ (Figure 3g–i). CaP coatings deposited using the three-electrode system are more uniform compared to coatings deposited using the two-electrode system. The obtained SEM results confirm the fact that the change in voltage/potential causes morphological changes in the deposited coatings, increasing microcracks, porosity, and surface development of the coatings. It can be expected that individual morphological features will influence various physicochemical properties of the coatings.

### 3.2. Thickness and Roughness of CaP Coatings

Table 2 describes the dependents of the thickness and roughness of the deposited CaP coatings on the applied electrode system and the voltage/potential values. It can be observed that the CaP coatings deposited in the three-electrode system are relatively thicker than those deposited in the two-electrode system. The thinnest coatings are deposited at −5 V/−5 V_SCE_ in both the two-electrode and three-electrode systems. The average roughness values do not exceed 3.50 µm, and the highest Ra values of 3.22 ± 0.21 µm and 3.30 ± 0.20 µm were obtained for coatings deposited at −5 V and −5 V_SCE_, respectively.

### 3.3. Energy Dispersive X-Ray Spectroscopy

**Figure 4:**
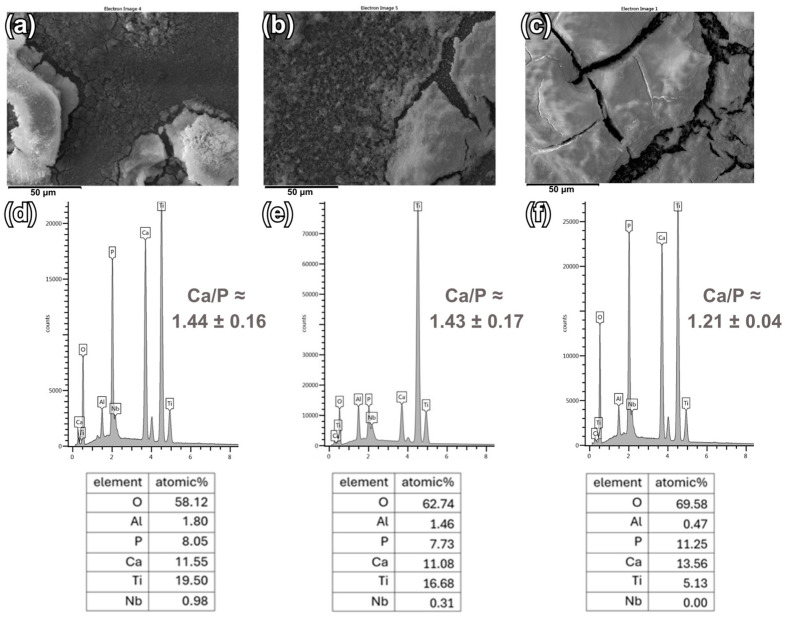
SEM images of the surface with the corresponding EDS spectra for the CaP coatings deposited at (**a**,**d**) −4 V; (**b**,**e**) −5 V; (**c**,**f**) −6 V.

**Figure 5:**
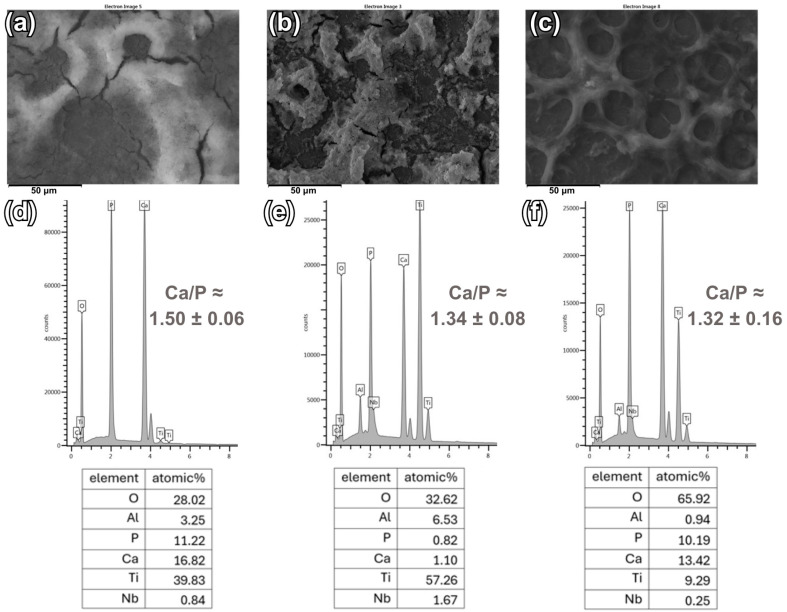
SEM images of the surface with the corresponding EDS spectra for the CaP coatings deposited at (**a**,**d**) −4 V_SCE_; (**b**,**e**) −5 V_SCE_; (**c**,**f**) −6 V_SCE_.

Figure 4 and Figure 5 show the analysis of the chemical composition of CaP coatings deposited using both electrode systems on Ti6Al7Nb substrates. The presented EDS spectra confirm the presence of elements in the coatings, i.e., Ca, P, and O, which are the main components of calcium phosphates. Moreover, in the case of relatively thinner coatings and non-uniform thickness of the deposited CaP coatings, elements originating from the Ti6Al7Nb substrate, such as Ti, Nb, or Al, can be noted. Trace amounts of alloying elements are not harmful to the human body and the presence of Ti in the chemical composition of the tested coatings does not have a negative effect on the surrounding tissues [62].

The atomic ratio of Ca and P in the coatings deposited in a two-electrode system (for coating deposited at: −4 V is 1.44 ± 0.16; at −5 V is 1.43 ± 0.17; at −6 V is 1.21 ± 0.04) are slightly lower than the atomic values of these elements in the coatings deposited in a three-electrode system (for coating deposited at: −4 V_SCE_ is 1.50 ± 0.06.; at −5 V_SCE_ is 1.34 ± 0.08; at −6 V_SCE_ is 1.32 ± 0.16). The molar Ca/P ratio of stoichiometric HAp is 1.67, but the resulting ratios Ca/P have lower values, which correspond to CDHA and DCPD [24,36,63]. A Ca/P value of 1.67 is considered important to maintain the chemical stability of HAp. It can be observed that changing the voltage and potential towards −6V/−6V_SCE_ causes a lower Ca/P ratio compared to the value of −4 V/−4 V_SCE_. Furthermore, the values obtained for coatings deposited at −4 V/−4 V_SCE_ are more similar to the Ca/P ratio of stoichiometric HAp. By analyzing the atomic values of the elements given in the tables, it can be seen that in the case of the coating deposited in the three-electrode system at a potential of −5 V_SCE_, the Ca and P values are much lower than the Ca and P values in the other coatings, which may indicate a relatively thin coating (Figure 5e).

### 3.4. Diffraction Measurements

**Figure 6:**
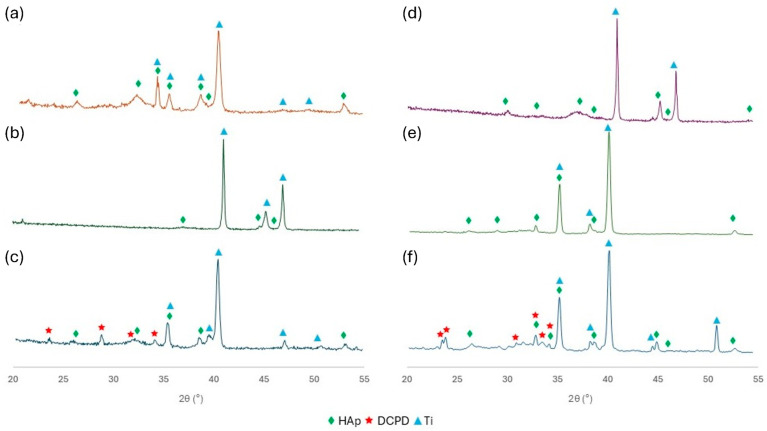
XRD patterns of the CaP coatings deposited in two-electrode system at (**a**) −4 V, (**b**) −5 V, (**c**) −6 V, and deposited in three-electrode system at (**d**) −4 V_SCE_, (**e**) −5 V_SCE_, (**f**) −6 V_SCE_ (♦ HAp; 🟊 DCPD; ▲ Ti).

XRD patterns of the CaP coatings electrochemically deposited on Ti6Al7Nb alloy using both electrode systems are shown in Figure 6. XRD patterns mainly indicate the occurrence of characteristic peaks for HAp (ICDD 055-0592), DCPD (ICDD 011-0293), and titanium (ICDD 002-2539) [64,65]. Intense reflections from the titanium substrate were detected in each XRD pattern and these reflections overlap the peaks corresponding to HAp. This phenomenon may be related to the relatively thin HAp coating. It can be observed that the HAp peaks for the coatings deposited at −5 V/−5 V_SCE_ are not sharp, which may indicate more amorphous and thinner coatings (Figure 6b,e). However, noticeably intense HAp peaks, defined as (002), (211), (112), (301), and (004) [66], occur in the CaP coating deposited at −4 V in the two-electrode system (Figure 6a). Coatings deposited at −4 V/−4 V_SCE_ are characterized by the most homogeneous phase structure compared to the others (Figure 6a,d). A small amount of DCPD, known as brushite, can also be identified in the coating deposited at −6 V/−6 V_SCE_ in both electrode systems (Figure 6c,f) [58]. This is probably due to changes in structure that occur with depth. The appearance of additional phases is often found in the literature describing the electrochemical deposition of CaP coatings [67,68]. The inhomogeneity of the XRD pattern may result from morphological deformations of the coatings, and the occurrence of micropores or cracks, which was confirmed by SEM observations.

### 3.5. Raman Spectroscopy

**Figure 7:**
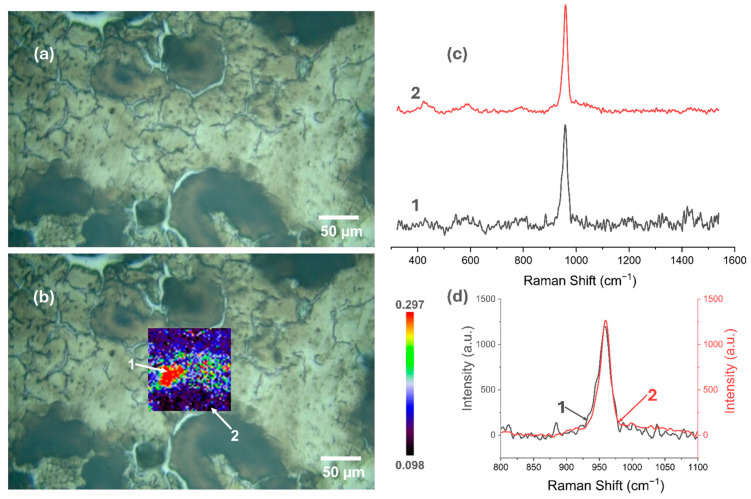
Raman spectroscopic analysis of CaP coating electrodeposited at −4 V in the two-electrode system: (**a**) view of the studied surface; (**b**) Raman maps of I_950_/I_963_; (**c**) view of spectra in the whole research range from selected locations in figure (**b**); (**d**) comparison of spectra in a narrow range from 800 to 1100 cm^−1^.

**Figure 8:**
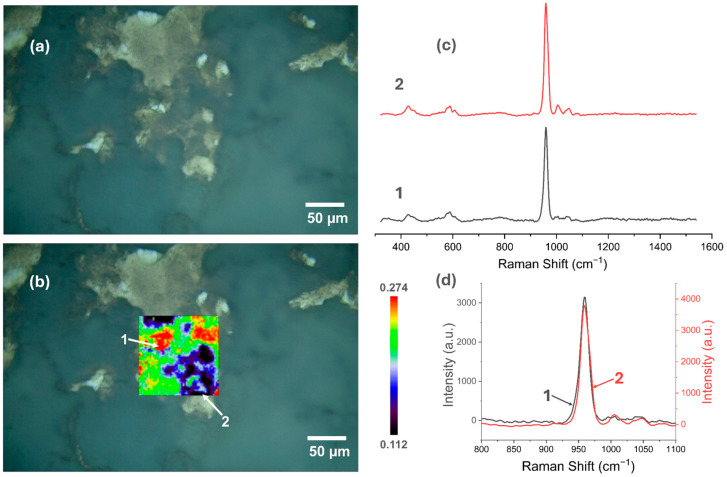
Raman spectroscopic analysis of CaP coating electrodeposited at −4 V_SCE_ in the three-electrode system: (**a**) view of the studied surface; (**b**) Raman maps of I_950_/I_963_; (**c**) view of spectra in the whole research range from selected locations in figure (**b**); (**d**) comparison of spectra in a narrow range from 800 to 1100 cm^−1^.

**Figure 9:**
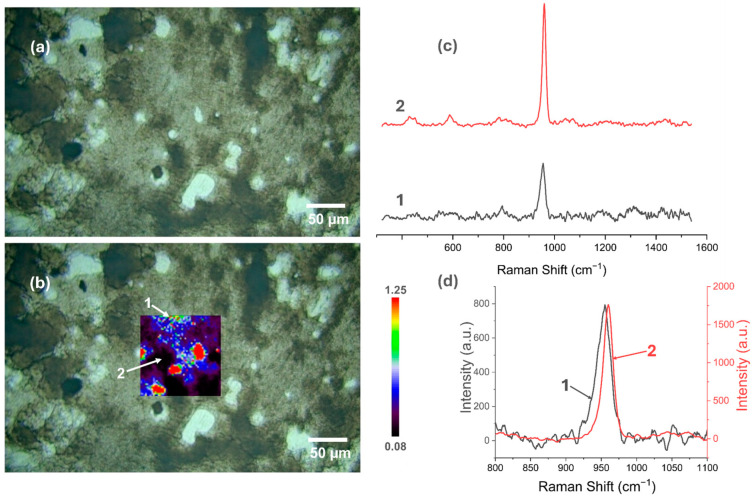
Raman spectroscopic analysis of CaP coating electrodeposited at −5 V in the two-electrode system: (**a**) view of the studied surface; (**b**) Raman maps of I_950_/I_963_; (**c**) view of spectra in the whole research range from selected locations in figure (**b**); (**d**) comparison of spectra in a narrow range from 800 to 1100 cm^−1^.

**Figure 10:**
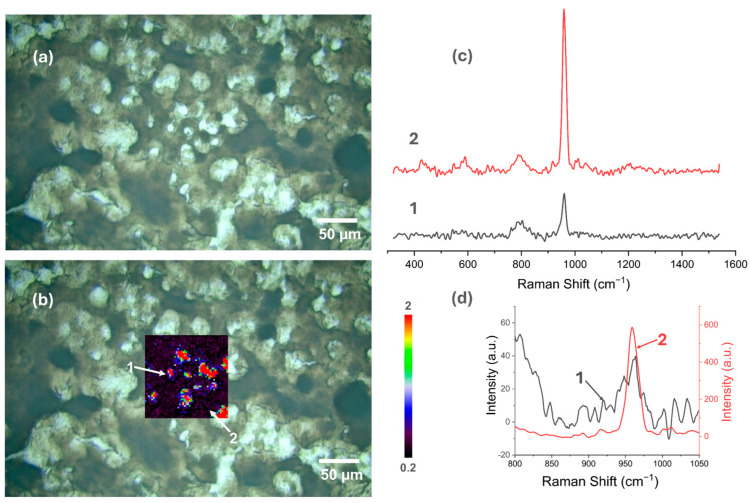
Raman spectroscopic analysis of CaP coating electrodeposited at −5 V_SCE_ in the three-electrode system: (**a**) view of the studied surface; (**b**) Raman maps of I_950_/I_963_; (**c**) view of spectra in the whole research range from selected locations in figure (**b**); (**d**) comparison of spectra in a narrow range from 800 to 1100 cm^−1.^

**Figure 11:**
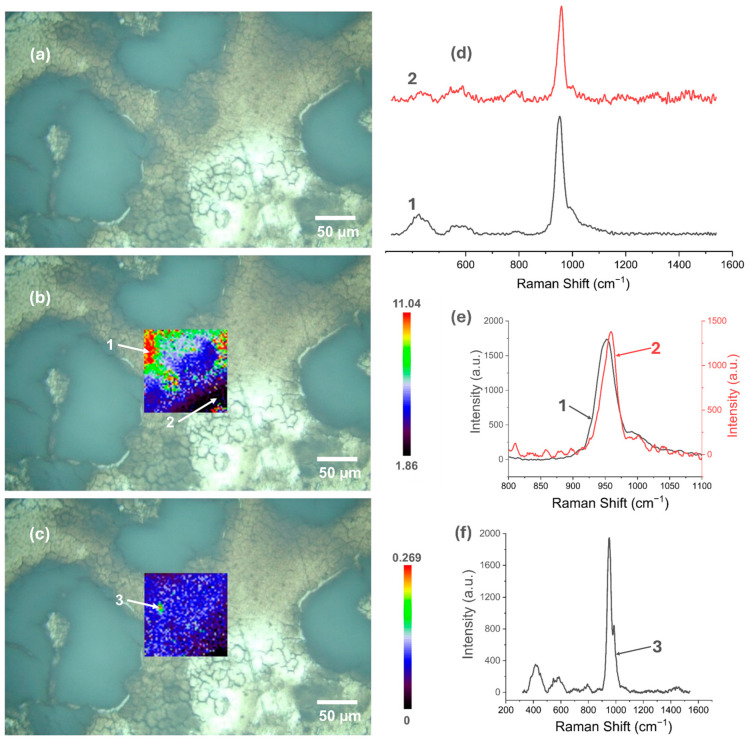
Raman spectroscopic analysis of CaP coating electrodeposited at −6 V in the two-electrode system: (**a**) view of the studied surface; (**b**) Raman maps of I_950_/I_963_; (**c**) analysis of the DCPD occurrence areas; (**d**) view of the spectra in the entire research range from selected locations in figure (**b**); (**e**) comparison of spectra in a narrow range from 800 to 1100 cm^−1^; (**f**) Spectrum from the selected location in figure (**c**).

**Figure 12:**
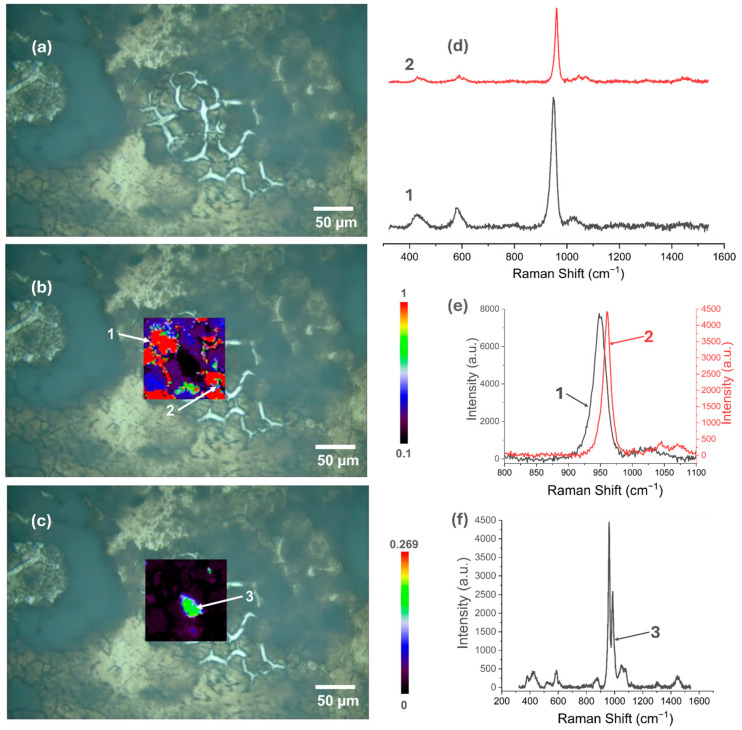
Raman spectroscopic analysis of CaP coating electrodeposited at −6 V_SCE_ in the three-electrode system: (**a**) view of the studied surface; (**b**) Raman maps of I_950_/I_963_; (**c**) analysis of the DCPD occurrence areas; (**d**) view of the spectra in the entire research range from selected locations in figure (**b**); (**e**) comparison of spectra in a narrow range from 800 to 1100 cm^−1^; (**f**) Spectrum from a selected location in figure (**c**).

Figure 7, Figure 8, Figure 9, Figure 10, Figure 11 and Figure 12 present the results of the work carried out using Raman spectroscopy. Most of the measurements indicate that the produced coatings have the chemical structure of typical HAp with the most intense peak around 962 cm^−1^ (ν1). In addition to this, peaks at about 431 cm^−1^ associated with (ν2) O-P-O oscillations, 581 and 593 cm^−1^ associated with (ν4) O-P-O oscillations, and at about 1072, 1046, and 1029 cm^−1^ for symmetric stretching (ν3) P-O oscillations can be observed on the obtained spectra. In addition to the HAp structure, in the coatings deposited at the most cathodic voltage parameters, the spectra indicating additional content in the DCPD structure (as in Figure S2) could be observed.

It is clear from the study that the most homogeneous chemical structure is found in coatings electrochemically deposited at −4 V (in the two-electrode system) and −4 V_SCE_ (in the three-electrode system). In the case of the two-electrode system, the map scale values range from 0.098 to 0.297 (Figure 7), while for the three-electrode system, the values range from 0.112 to 0.274 (Figure 8). Thus, the range of obtained minimum and maximum values of the I_950_/I_963_ ratio is very narrow. The homogeneity of the chemical structure of the tested coatings is confirmed by comparisons of the Raman spectra (Figure 7c,d and Figure 8c,d). The presented plots of spectra from the extremes describing the values of the I_950_/I_963_ ratio are very similar to each other.

Applying a more cathodic potential of −5 V, results in changes in the chemical structure of the produced coatings, which are visible, including by an increase in the value scale maps (for the two-electrode system to a value of 1.25, while for the three-electrode system to a value of 2). The coatings produced under these parameters become more amorphous (the maximum value of the I_950_/I_963_ ratio is at least three times higher than for the modification at −4 V/−4 V_SCE_), and it is characteristic that the areas with the highest I_950_/I_963_ ratio are in places where the optical microscopy images show a clear surface brightening and consequently a decrease in the intensity of the obtained spectra (Figure 9c,d and Figure 10c,d).

In the coatings deposited at −6 V at the two-electrode system and at −6 V_SCE_ at the three-electrode system, there are visible areas where the coating is a mixture of HAp and DCPD (Figure 11c,f and Figure 12c,f). When using a two-electrode system, the amorphization of the coating increases (the map value scale increases to 11.04), but in this case, no large differences in the signal intensity from the places with the highest and lowest I_950_/I_963_ ratio were observed. For the three-electrode system and the potential of −6 V_SCE_, the change in the map scale value is from 0.1 to 1, which indicates a better homogeneity of the chemical structure of the produced coating compared to the two-electrode system.

### 3.6. Corrosion Study

Several factors, including the homogeneity, continuity, phase composition, and crystallinity of biomedical coatings—attributes influenced by the deposition conditions—determine the corrosion properties of these coatings.

**Figure 13:**
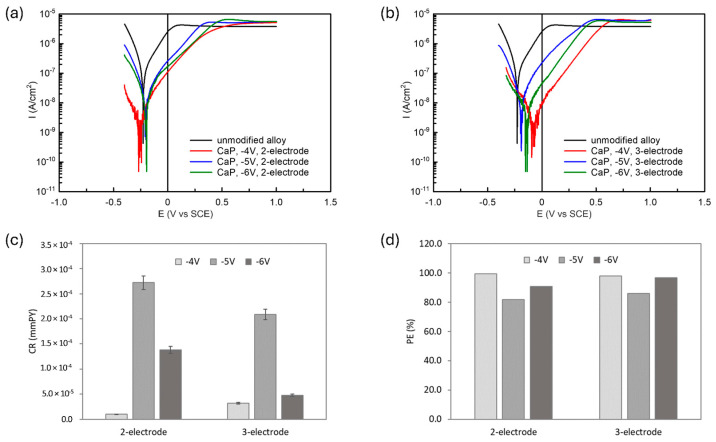
Potentiodynamic polarization curves for unmodified Ti6Al7Nb alloy and CaP-coated surfaces obtained using (**a**) two-electrode and (**b**) three-electrode electrodeposition systems. Calculated (**c**) corrosion rate of CaP-coated surfaces and (**d**) protection efficiency of the coatings in PBS solution.

Figure 13 illustrates the polarization behavior of both unmodified and CaP-coated Ti6Al7Nb alloy samples in PBS solution. All samples exhibit passivity during anodic polarization up to 1 V; however, the electrodeposited CaP coatings significantly alter the corrosion properties of the alloy under investigation. The kinetic parameters of the electrochemical corrosion reaction, including the corrosion potential (E_cor_), corrosion current density (I_cor_), and the anodic and cathodic Tafel slopes (b_a_ and b_c_) were determined by the Tafel extrapolation method and summarized in Table 3. These values of I_cor_, b_a_, and b_c_ were then used to calculate the polarization resistance (R_p_) according to the Stern-Geary equation (Equation (1)) [69]:

(1) $$I_{cor}=\frac{b_{a}b_{c}}{2.303\left(b_{a}+b_{c}\right)R_{p}}$$

In accordance with ASTM G102-89 standard, the corrosion rate (C_R_) was estimated using the following equation (Equation (2)) [70]:

(2) $$C_{R}=K_{1}\frac{I_{cor}}{d}EW$$

where K_1_ is a constant (for C_R_ given in mmPY it equals 3.27·10^−3^ mm g/µA cm year), d represents the alloy density (4.52 g/cm^3^ for Ti6Al7Nb), and EW is the equivalent weight of the alloy (assumed to be 12.04).

The corresponding values for R_p_ and C_R_ are provided in Table 3.

The data presented in Figure 13 and Table 3 show that all the applied CaP coatings reduced I_cor_ values of the Ti6Al7Nb alloy, indicating enhanced corrosion resistance in PBS solution. The most effective protective performance was observed for CaP coatings electrodeposited at −4 V, both in the two- and three-electrode systems. Conversely, the weakest corrosion resistance was noted in the samples with CaP coatings electrodeposited at −5 V, irrespective of the electrode system used.

The diminished corrosion resistance of the coatings deposited at −5 V is attributed to two main factors. In the two-electrode system, the coatings formed at −5 V were thinner, providing less effective protection. In the three-electrode system, discontinuities were observed in the coating, which likely increased corrosion susceptibility. These structural weaknesses are critical, as they undermine the coating’s ability to form a continuous passive layer, essential for corrosion protection.

In contrast, the CaP coatings deposited at −4 V exhibited superior corrosion resistance, evidenced by the highest R_p_ and the lowest CR values among the tested samples. This enhanced performance, irrespective of the electrode system used, is likely due to the formation of more crystalline HAp coatings under these electrodeposition conditions. The improved crystallinity provides a more effective barrier against corrosion. These observations are further supported by the protection efficiency (PE) values (calculated based on Equation (3)), which show higher protection efficiency for crystalline HAp coatings deposited at −4 V (PE ranging from 98% to 99%) than for mixed-CaP (HAp with DCPD) coating deposited at −6V in the two-electrode system (PE ca. 91%).

(3) $$PE=\left(1-\frac{I_{cor,c}}{I_{cor,s}}\right)\cdot 100\%$$

where I_cor,c_ and I_cor,s_ are the corrosion current density of the coated and bare substrate, respectively [52].

The deposition of CaP coatings at less negative voltage/potential, such as −4 V/−4 V_SCE_, creates favorable electrochemical conditions for the formation of stoichiometric HAp coatings with fewer defects and higher crystallinity. In contrast, more negative voltage/potential (e.g., −5 V/−5 V_SCE_ or −6 V/−6 V_SCE_) leads to excessive hydrogen evolution during the electrodeposition process, resulting in coating discontinuities and decreased crystallinity. Furthermore, these more negative voltages/potentials promote the formation of undesired phases, such as DCPD, which compromise the stoichiometry and protective properties of the HAp coating.

## 4. Conclusions

CaP coatings were successfully electrochemically deposited on a Ti6Al7Nb alloy substrate using both two-electrode and three-electrode systems.

It can be concluded that the electrode system used and the value of voltage/potential applied in the electrodeposition process of CaP coatings have an impact on the morphology of the deposited coatings. Nevertheless, in the case of a three-electrode system, the morphology of the coatings is more compact. EDS studies confirmed the presence of basic elements in CaP coatings. Changing the voltage/potential to a more cathodic causes a decrease in the Ca/P value and the ratio is close to DCPD, which was also confirmed by XRD studies and Raman spectroscopy.

Analysis of XRD and Raman spectroscopy results allows us to state that the chemical structure and occurrence of individual CaP phases change depending on the applied voltage/potential parameters. The most structurally homogeneous coatings were deposited using −4 V and −4 V_SCE_.

The results of corrosion tests indicate that electrodeposition at −4 V/−4 V_SCE_ provides the optimal conditions for producing CaP coatings with superior corrosion resistance, with high R_p_ and low CR values, attributed to a high level of the crystalline HAp phase in the hydroxyapatite coating. The findings highlight the importance of selecting appropriate deposition potentials to control the morphology and phase composition of the coatings, ensuring maximum corrosion protection for biomedical applications.

## Figures and Tables

**Table 1:** Parameters of electrochemical processes.

	Voltage/Potential	Deposition Time
two-electrode system	−4 V; −5 V; −6 V	2 h
three-electrode system	−4 V_SCE_; −5 V_SCE_; −6 V_SCE_	

**Table 2:** Thickness and average surface roughness values (R_a_) of CaP coatings.

Type of the Electrode System	Applied Voltage/Potential	Thickness (µm)	Average Surface Roughness, R_a_ (µm)
two-electrode system	−4 V	3.8 ± 0.5	1.19 ± 0.15
	−5 V	1.7 ± 0.3	3.22 ± 0.21
	−6 V	2.5 ± 0.4	2.91 ± 0.19
three-electrode system	−4 V_SCE_	17.5 ± 0.6	2.66 ± 0.08
	−5 V_SCE_	7.5 ± 0.5	3.30 ± 0.20
	−6 V_SCE_	16.7 ± 0.7	2.67 ± 0.27

**Table 3:** Electrochemical parameters for uncoated and CaP-coated Ti6Al7Nb alloy surfaces in PBS solution.

Sample	b_a_ (mV)	b_c_ (mV)	E_cor_ (V)	I_cor_ (A/cm^2^)	R_p_ (MOhm cm^2^)	C_R_ (mmPY)	PE (%)
**unmodified alloy**	209 ± 18	113 ± 10	−0.227 ± 0.012	(1.72 ± 0.09)·10^−7^	0.19 ± 0.01	(1.50 ± 0.08)·10^−3^	-
two-electrode set-up							
**CaP, −4 V**	98 ± 9	91 ± 8	−0.263 ± 0.014	(1.13 ± 0.06)·10^−9^	18.10 ± 1.17	(9.87 ± 0.49)·10^−6^	99.3
**CaP, −5 V**	247 ± 15	121 ± 11	−0.210 ± 0.018	(3.12 ± 0.16)·10^−8^	1.13 ± 0.08	(2.72 ± 0.14)·10^−4^	81.8
**CaP, −6 V**	155 ± 10	129 ± 12	−0.197 ± 0.012	(1.58 ± 0.08)·10^−8^	1.93 ± 0.12	(1.38 ± 0.07)·10^−4^	90.8
three-electrode set-up							
**CaP, −4 V_SCE_**	205 ± 15	154 ± 13	−0.098 ± 0.022	(3.68 ± 0.18)·10^−9^	10.41 ± 0.75	(3.21 ± 0.16)·10^−5^	97.9
**CaP, −5 V_SCE_**	198 ± 14	121 ± 12	−0.194 ± 0.015	(2.40 ± 0.12)·10^−8^	1.36 ± 0.11	(2.09 ± 0.10)·10^−4^	86.0
**CaP, −6 V_SCE_**	146 ± 9	169 ± 12	−0.140 ± 0.012	(5.47 ± 0.27)·10^−9^	6.22 ± 0.44	(4.78 ± 0.24)·10^−5^	96.8

## Data Availability

The original contributions presented in this study are included in the article/Supplementary Materials. Further inquiries can be directed to the corresponding author.

## Supplementary Materials

The following supporting information can be downloaded at: https://www.mdpi.com/article/10.3390/ma18030539/s1. Figure S1. The deconvolution process for the selected spectrum; Figure S2. Raman spectra of DCPD; Figure S3. Raman spectra of HAp.
