# Supplementary material for: Impact of the Applied Electrode System on Properties of Electrodeposited Calcium Phosphate Coatings
Source: Materials (Basel). 2025 Jan 24;18(3):539. doi: 10.3390/ma18030539 (PMC11818842; doi:10.3390/ma18030539)
Supplement: Supplementary file 1 [file materials-18-00539-s001.zip › materials-3367843-supplementary.pdf]

materials

MDPI

Electronic Supplementary Information

Impact of the Applied Electrode System on Properties of Electrodeposited Calcium Phosphate Coatings

Klaudia Iwaniak, Witold Kaczorowski, Barbara Burnat, Jacek Grabarczyk

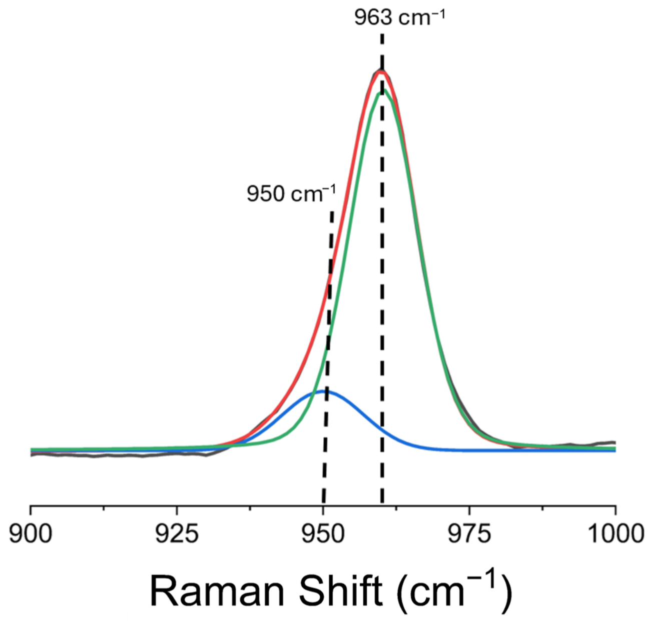

Figure S1. The deconvolution process for the selected spectrum.

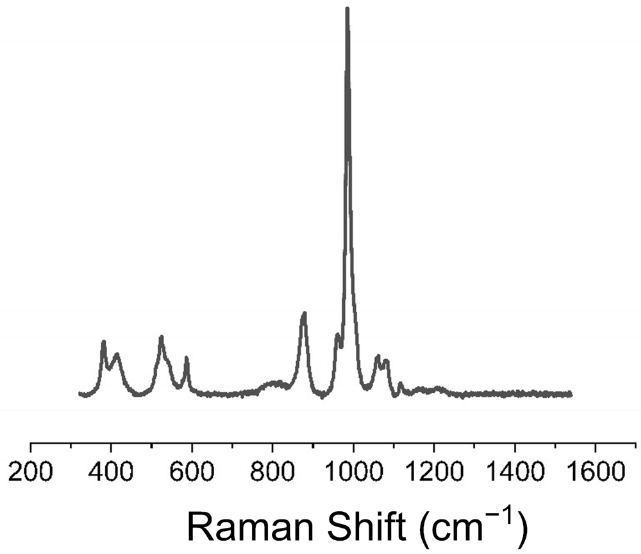

Figure S2. Raman spectra of DCPD.

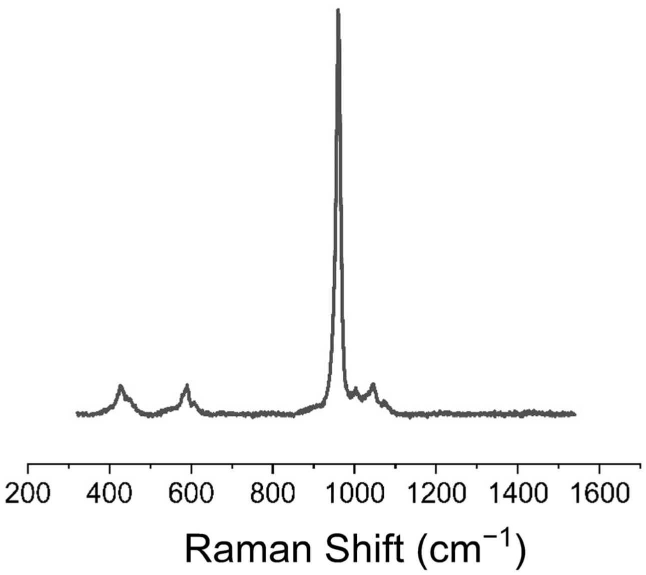

Figure S3. Raman spectra of HAp.
